# Sonographic Anatomy and Imaging of the Triangular Fibrocartilage Complex and the Distal Radio‐Ulnar Joint

**DOI:** 10.1002/ajum.70012

**Published:** 2025-07-01

**Authors:** Craig Winnett, Michelle Fenech

**Affiliations:** ^1^ School of Health, Medical and Applied Sciences, College of Clinical Sciences Central Queensland University Brisbane Queensland Australia; ^2^ I‐MED Radiology Brisbane Queensland Australia; ^3^ Department of Medical Imaging Princess Alexandra Hospital Brisbane Queensland Australia; ^4^ Department of Medical Imaging Royal Brisbane and Women's Hospital Herston, Brisbane Queensland Australia

**Keywords:** disc proper, distal radio‐ulnar ligaments, ECU sub‐sheath, meniscus homologue, musculoskeletal ultrasound, ulnar‐sided wrist pain

## Abstract

**Introduction:**

Ulnar‐sided wrist pain is a common clinical problem, which can be difficult to diagnose, manage and treat. Ultrasound imaging can be used to identify structural changes which may contribute to the pain. Knowledge of the associated relative anatomy and intertwining structures which form the triangular fibrocartilage complex (TFCC) and distal radio‐ulnar joint (DRUJ), sonographic techniques to image these structures and their normal and abnormal appearances can be underappreciated and are required.

**Methods:**

A literature search was conducted to review the current knowledge related to sonographic assessment of the TFCC and DRUJ.

**Results:**

The intertwining components of the DRUJ and TFCC which can be demonstrated sonographically are unpacked with clear supporting figures and videos.

**Discussion:**

The components of the TFCC, which include the disc proper, meniscus homologue, extensor carpi ulnaris (ECU) sub‐sheath, palmar‐sided ulno‐carpal ligaments and the palmar and dorsal distal radio‐ulnar ligaments, can all be individually demonstrated sonographically, in addition to structures which comprise the DRUJ. Normal sonographic images are presented.

**Conclusion:**

Improved understanding of the sonographic anatomy, technique and normal imaging appearances of ulnar‐sided wrist structures can enhance the quality of imaging and subsequently the diagnosis of structural causes of pain, which can guide patient management.

## 1 Introduction

Ulnar‐sided wrist pain can be referred to as the ‘back pain’ or ‘black box’ of the wrist due to the complex anatomy of this small anatomic region, making clinical diagnosis of the cause of pain difficult [1]. Causes of ulnar‐sided wrist pain can include triangular fibrocartilage complex (TFCC) tears, distal radio‐ulnar joint (DRUJ) arthrosis or instability and extensor carpi ulnaris (ECU) tendinopathy, tears and/or subluxation [2]. Further differential diagnoses can include ulnar styloid fractures, hook of hamate fracture, hamate facet arthrosis, piso‐triquetral arthropathy, Keinbock disease (avascular necrosis of the lunate), luno‐triquetral injuries and instability, flexor carpi ulnaris (FCU) and flexor carpi radialis (FCR) tendinopathy, ulnar nerve (UN) compression (including its branches) and ulnar impaction syndrome [3]. Clinical diagnosis of ulnar wrist pain is often difficult because pathologies or injuries may be co‐existent [4]. Ultrasound imaging is frequently requested to identify potential structural change of the TFCC and DRUJ, and guide injections for pain relief. Sonographic imaging and assessment of the ulnar side of the wrist can be difficult due to the complex nature of the intertwining of structures which comprise the TFCC and DRUJ and the complex injury patterns. Most literature which unpacks the structural components of the TFCC and DRUJ for imaging relates to magnetic resonance imaging (MRI). The deeper and central aspects of the TFCC do require MR imaging to identify/exclude tears of this region, however, ultrasound can play a role in imaging peripheral tears. To undertake a sonographic assessment of the TFCC and DRUJ, the relative anatomy of the sub‐structures which comprise the TFCC and DRUJ, the sonographic technique to image them and the associated normal sonographic appearances must be firstly understood and appreciated.

## 2 Sonographic Considerations for Imaging the Ulnar Side of the Wrist

**FIGURE 1:**
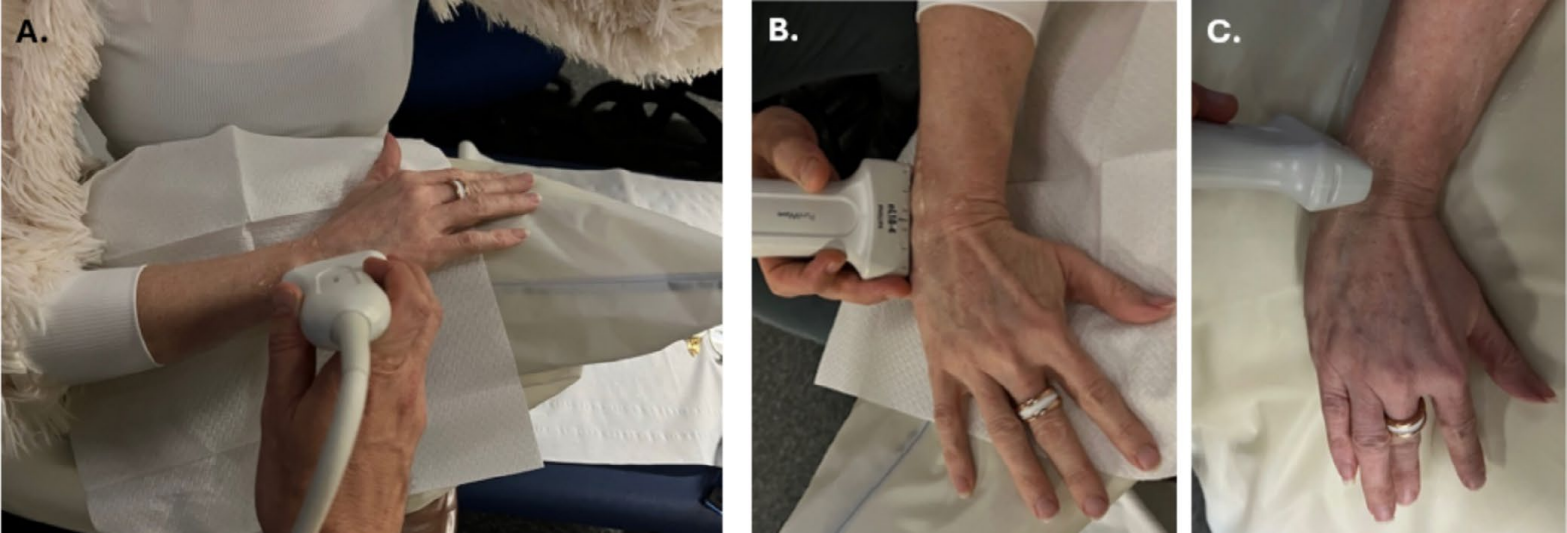
Positioning of the wrist for sonographic assessment of the ulnar aspect. (A) Patient is seated, hand pronated, forearm elevated to shoulder height, with a pillow supporting the forearm allowing long axis imaging from a dorso‐ulnar approach. (B) Long axis imaging with the hand radially deviated to open the ulno‐carpal space. (C) Short axis imaging with the hand radially deviated.

Ultrasound imaging of the TFCC and DRUJ can provide high spatial resolution imaging and has the advantage over magnetic resonance (MR) due to the ability to dynamically assess the wrist. For sonographic assessment of the ulnar side of the wrist, imaging from the dorsal, palmar and ulnar aspects is required. To access the ulnar aspect of the wrist with an ultrasound transducer, the patient can be seated, facing towards the sonographer. The patient's forearm is positioned, resting and supported on a cushion or pillow at their shoulder level. The patient can pronate their hand (palm down) or raise the ulnar aspect of their hand and wrist towards the ceiling. This allows wrist flexion and extension and ulnar and radial deviation to be performed while imaging (Figure 1).

A combination of high frequency, small and large footprint transducers, with generous quantities of acoustic gel can be used. The wider footprint transducer allows relative bony structures of the ulnar side of the wrist and DRUJ to be appreciated. The smaller footprint transducer assists in maintaining contact during dynamic manoeuvres and imaging around the bony prominence of the ulnar styloid.

## 3 Anatomy and Sonographic Imaging of the Distal Radio‐Ulnar Joint

**FIGURE 2:**
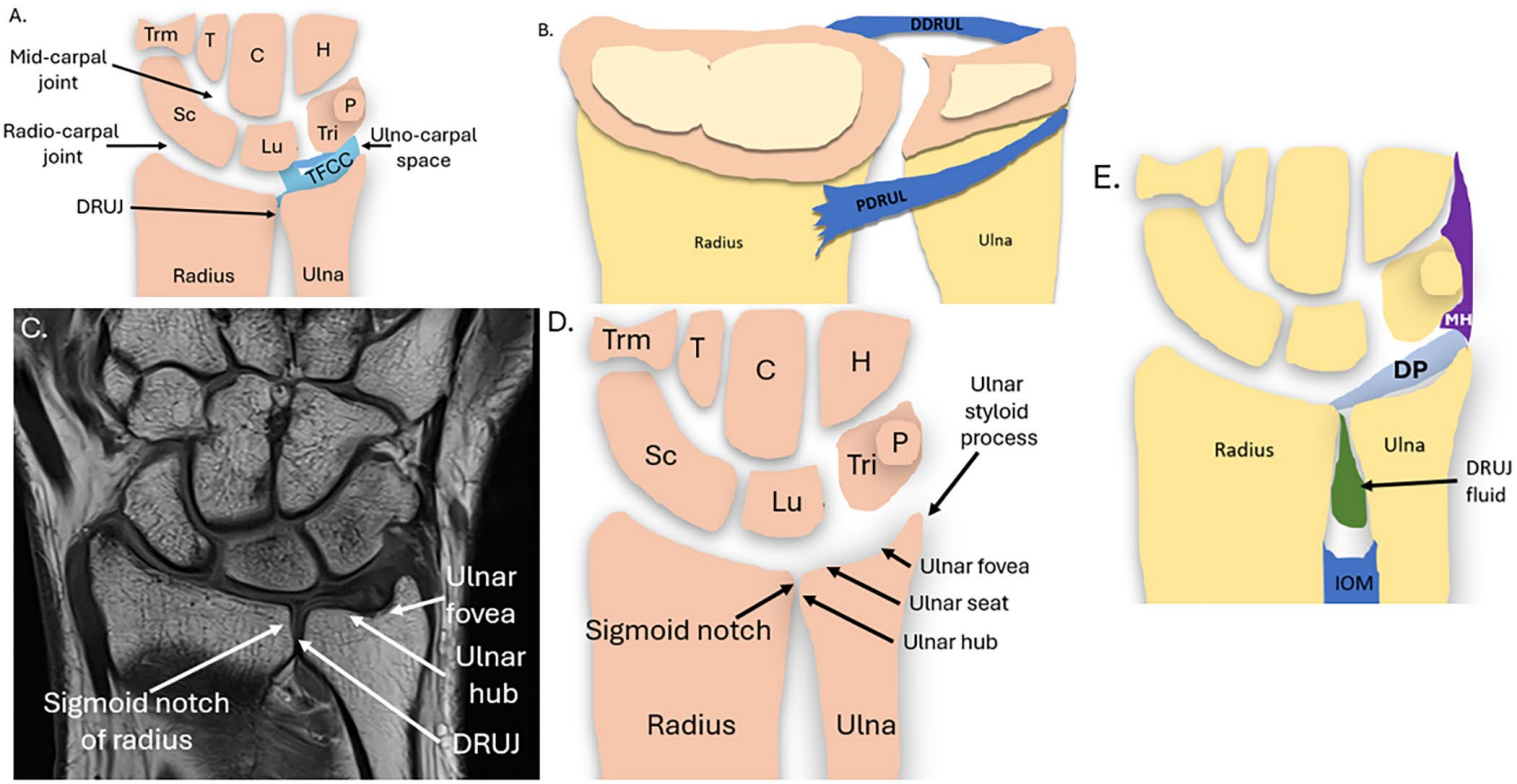
The anatomy of the wrist joints including the distal radio‐ulnar joint (DRUJ) and the ulno‐carpal space. (A) Outline of wrist joints. (B) The palmar and dorsal distal radioulnar ligaments of the DRUJ. (C) Coronal magnetic resonance image of the wrist. (D) Components of the ulnar head. (E) Proximal extension of DRUJ fluid. C, capitate; DDRUL, dorsal distal radio‐ulnar ligament; DP, disc proper; H, hamate; IOM, interosseous membrane; Lu, lunate; MH, meniscus homologue; P, pisiform; PDRUL, palmar distal radio‐ulnar ligament; Sc, scaphoid; T, trapezoid; TFCC, triangular fibrocartilage complex; Tri, triquetrum; Trm, trapezium.

The wrist is formed by three main joints: the DRUJ, radiocarpal joint (RCJ) and mid‐carpal joint (MCJ); these joints, in their normal state, do not communicate with each other. Some literature refers to the wrist containing an ‘ulnocarpal joint’ however, this is best considered a ‘space’, which contains the TFCC. The DRUJ is the distal articulation between the radius and ulna. As a pivot joint it allows forearm supination and pronation of the hand, in conjunction with grasping functions and also acts as a major weight bearing joint, distributing forces across the forearm [5]. The articulating surfaces of the DRUJ include the sigmoid notch of the radius and the ulnar head. The ulnar head consists of the ulnar hub, seat (dome), fovea and the styloid process which need to be appreciated on imaging [5]. The ulnar hub, directly articulates with the sigmoid notch of the radius at the DRUJ. The ulnar seat (dome) is directed distally towards the lunate and a common site for degenerative chondromalacia changes. The ulnar fovea and styloid process serve as a critical attachment sites for multiple capsuloligamentous structures of the TFCC that sit within the ulnocarpal space [5] (Figure 2).

The DRUJ is inherently unstable, relying heavily on intrinsic and extrinsic soft tissue structures [5]. The TFCC is a well‐defined anatomical entity which functions primarily to stabilise the distal radio‐ulnar joint (DRUJ) and act as a shock absorber across the ulno‐carpal space [2]. The TFCC consists of the dorsal and palmar distal radio‐ulnar ligaments (DRULs) which act in concert to stabilise the DRUJ depending on the wrist position [5]. Hence, the TFCC and DRUJ, due to being intertwined, cannot be sonographically assessed in isolation, but rather, must be assessed concurrently.

### 3.1 Extrinsic Stabilisers of the DRUJ

Proximal to the DRUJ, the pronator quadratus muscle and interosseous membrane of the distal anterior forearm are DRUJ extrinsic stabilisers offering a lesser contribution to the joint's stability [5].

#### 3.1.1 Pronator Quadratus Muscle

**FIGURE 3:**
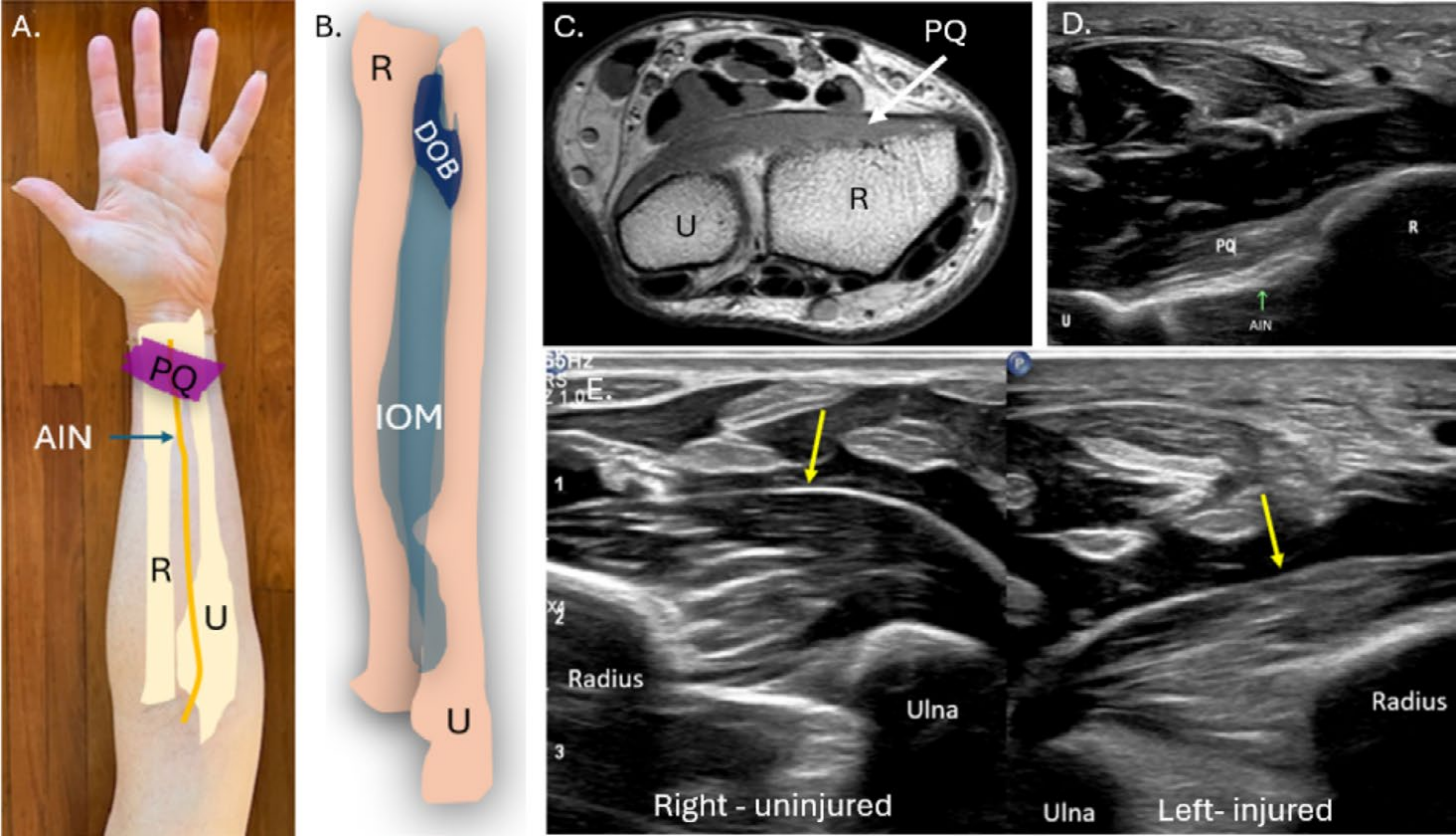
(A) Position of the pronator quadratus (PQ) muscle and the anterior interosseous nerve (AIN) in the anterior forearm. (B) The interosseous membrane (IOM) runs between the radius (R) and ulna (U). The distal end of the IOM is the thickened distal oblique bundle (DOB) which attaches to the dorsal rim of the sigmoid notch of the radius. (C) Axial magnetic resonance (MR) image of the distal forearm demonstrating the PQ muscle. (D) Short axis sonographic image of the distal anterior forearm, demonstrating the PQ muscles (yellow arrow) and the AIN deep to it. (E) Comparison short axis images of the distal anterior forearm, demonstrating the PQ muscle in uninjured and injured limbs. The left forearm and radius had been fractured and the AIN was entrapped by fracture fragments causing denervation and subsequent atrophy and increased echogenicity of the PQ muscle.

The pronator quadratus muscle (PQ) is a trapezoidal‐shaped muscle that spans between the distal anterior (palmar) surfaces of the radius and ulna, consists of superficial and deep heads and produces forearm pronation [5]. It is innervated by the anterior interosseous nerve (AIN), a branch of the median nerve, which sits deep to the PQ [6]. The AIN also provides sensory innervation to the DRUJ [6]. Intimate with the ulnar and palmar aspects of the DRUJ capsule, the PQ tenses the capsule to prevent the redundant pouch from being interposed within the DRUJ on forearm rotation and restricts dorsal translation of the radius relative to the ulna [5]. Although primary disorders affecting the PQ are uncommon, it should still be imaged and assessed when exploring structural causes of ulnar‐sided wrist pain (Figure 3).

#### 3.1.2 Interosseous Membrane

The interosseous membrane (IOM) is a broad, sheet‐like connective tissue of varying thickness throughout that courses between the radius, extending distally and obliquely to the ulna and can contain accessory bands [5]. The IOM prevents proximal migration and dorsal translation of the radius, maintaining longitudinal stability between these bones and plays a role in DRUJ stability [5]. It is thickened at its most distal margin, termed the distal oblique bundle (DOB) [7]. The DOB inserts distally onto the dorsal inferior rim of the sigmoid notch of the radius, allowing it to assist in stabilising the DRUJ [5]. The IOM should be assessed in cases of forearm fractures, particularly Galeazzi fractures of the mid‐ to distal radius, which are associated with IOM injury and DRUJ dislocation or subluxation [7].

#### 3.1.3 Sonographic Imaging of the DRUJ

Ultrasound can be used to image the DRUJ, evaluate its integrity and identify bony change, synovitis and instability [5]. Traumatic, overuse and degenerative disorders of the wrist are the most common causes of DRUJ dysfunction [5]. Osteophytes at the DRUJ are correlated with degenerative changes, and severe tears of the TFCC can cause DRUJ instability, which leads to progressive arthritis and dysfunction [3]. The DRUJ should be sonographically assessed with the hand in both pronation and maximal supination [3]. Dynamic sonographic imaging of the DRUJ, including medial to lateral and proximal to distal sweeps, should be conducted. When imaging the DRUJ, assessment of the distal forearm, including to the level of the pronator quadratus muscle, is important, as DRUJ fluid, when present, will extend proximally and can be defined and measured in short and long axis planes (Video 1).

**VIDEO 1:** Short and long axis imaging of the distal radio‐ulnar joint (1 min 20 s). Video content can be viewed at https://onlinelibrary.wiley.com/doi/10.1002/ajum.70012

## 4 Anatomy and Sonographic Imaging of the Triangular Fibrocartilage Complex

**FIGURE 4:**
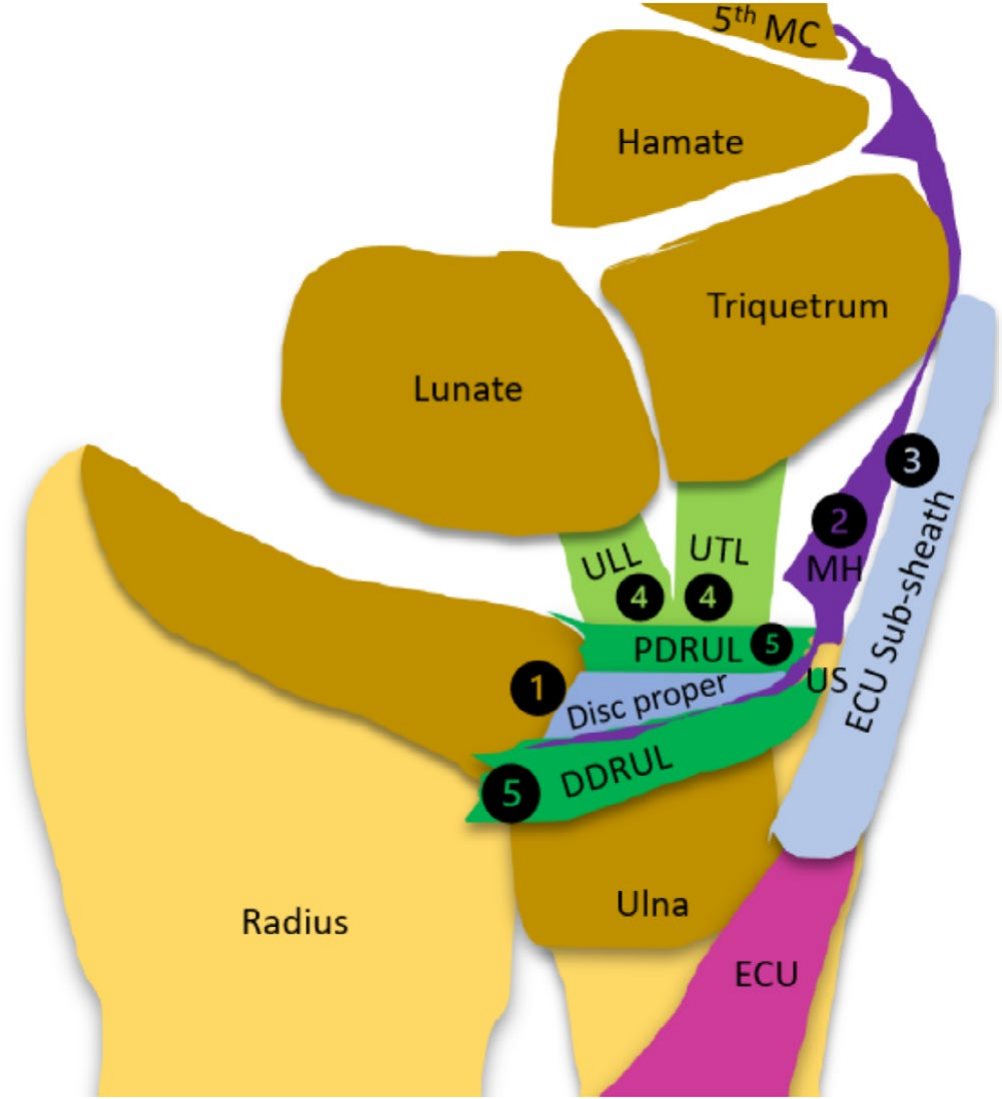
Components of the triangular fibro‐cartilage complex (TFCC) viewed from a dorsal approach which include (1) disc proper (full extent not shown in this diagram); (2) meniscus homologue (MH); (3) extensor carpi ulnaris (ECU) tendon sub‐sheath; (4) palmar ulno‐carpal ligaments: ulno‐lunate ligament (ULL) and ulno‐triquetral ligament (UTL); and (5) dorsal distal radio‐ulnar ligament (DDRUL) and palmar distal radio‐ulnar ligament (PDRUL). MC, metacarpal; TRIQ, triquetrum; ULN, ulna; US, ulnar styloid process.

The TFCC is an intricate fibrocartilage–ligamentous complex which has three main functions: (1) stabilises the DRUJ, (2) stabilises the ulno‐carpal space and (3) acts as a cushion and shock absorber and transmits load through the wrist during different wrist positions [8, 9, 10]. The components comprising the TFCC are variably described with inconsistent nomenclature, which causes confusion. There are however five main components of the TFCC which are most consistently outlined [10, 11]. These include the following: (1) disc proper; (2) meniscus homologue; (3) extensor carpi ulnaris (ECU) tendon sub‐sheath; (4) two palmar sided ulno‐carpal ligaments: the ulno‐lunate ligament (ULL) and the ulno‐triquetral ligament (UTL); and (5) two distal radio‐ulnar ligaments: the dorsal distal radio‐ulnar ligament (DDRUL) and the palmar distal radio‐ulnar ligament (PDRUL) [11]. These main TFCC components and relative structures and their sonographic appearances are unpacked in this paper (Figure 4).

### 4.1 Disc Proper

The disc proper (DP) of the TFCC consists of triangular‐shaped fibrocartilage and is also referred to as the ‘fibrocartilage disc’. Cartilage is a special connective tissue characterised by a cellular component immersed within an extracellular matrix composed of ground substance (polysaccharides), a fibrillar component (fibrous proteins) and interstitial fluid (mainly water) [12]. There are three main types of cartilage in the body, which include hyaline (articular) cartilage, fibrocartilage, and elastic cartilage; each differs in their composition, locations and function [12]. Hyaline cartilage is present at the articulating surfaces of synovial joints, in the trachea and at the connection between the ribs and sternum. Fibrocartilage has a matrix rich in densely braided collagen fibres that make it highly resistant to compression; furthermore, chondrocytes are low in number and aligned with the thick collagen fibres [12]. The intervertebral discs of the spine, the pubic symphysis, the menisci of the knee and the tendon–bone interfaces at entheses are all composed of fibrocartilage. The DP can also be termed the articular or articulate disc, but this term can be confusing, as it may be interpreted that the disc contains articular cartilage; for this reason, in this paper, it is referred to as the DP.

The DP attaches the radius to the ulna and facilitates smooth motion of the wrist by stabilising the DRUJ [13]. The DP extends between three attachment sites: (1) the sigmoid notch of the radius, (2) the ulnar fovea and (3) the ulnar styloid process. The DP fans out from the sigmoid notch of the radius initially as a single strong band, called the radial attachment (RA) [10]. The DP then divides into two bands (lamina), a proximal and distal lamina, as it extends towards the ulnar side of the wrist [13]. The ulnar side or attachments of the DP can be collectively termed the ‘triangular ligament’ as it indicates the convergence of two laminae from the DP [14]. The proximal lamina of the DP courses from the sigmoid notch of the radius, over the ulnar seat and attaches to the ulnar fovea, whereas the distal lamina attaches to the distal aspect of the ulnar styloid process [10].

**FIGURE 5:**
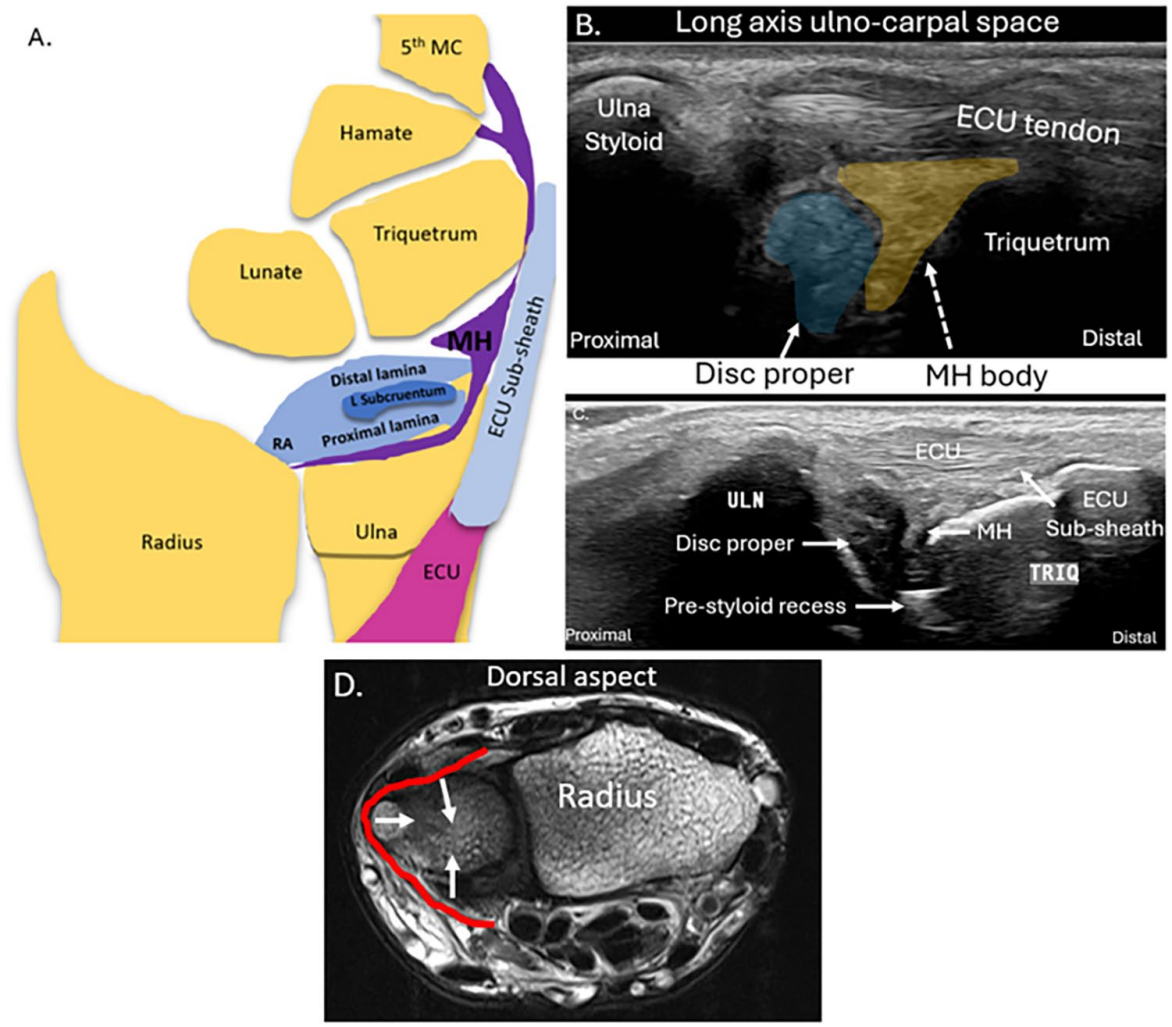
(A) Diagram of the disc proper of the triangular fibrocartilage complex (TFCC) demonstrating its extension and its constituent parts: (1) radial attachment (RA), (2) proximal lamina, (3) distal lamina and (4) ligamentum subcruentum (L = ligamentum). (B) Long axis sonographic image of the TFCC outlining the difference between the disc proper and body of the meniscus homologue. (C) Long axis sonographic image of the ulnocarpal space indicating the position of the disc proper. (D) Axial magnetic resonance image of the wrist outlining the arterial interconnections (red line) between the dorsal and palmar branches of the anterior interosseous arteries and direction of perfusion of the disc proper. Dist, distal aspect of image; ECU, extensor carpi ulnaris; MC, metacarpal; MH, meniscus homologue; Prox, proximal aspect of image; TRIQ, triquetrum; ULN, ulna; US, ulnar styloid process.

Lying between the DP proximal and distal lamina at the ulnar fovea, is a small wedge‐shaped channel of fibrovascular connective tissue called the ‘ligamentum subcruentum’; at the ulnar fovea, blood vessels pass from the bone to the subcruentum [5, 15]. The ligamentum subcruentum is variably described to extend to anteriorly and posteriorly to the PDRUL and DDRUL at the ulnar fovea [5]. Although the ligamentum subcruentum can be identified on MRI, and has high signal on fluid sensitive sequences due to its rich vascularity, due to its deep location in the curvature of the ulnar fovea, it is not usually sonographically visible as a separate entity [10]. Hence, injuries to this region may not be appreciated sonographically (Figure 5).

The periphery (superficial portions) of the DP is relatively vascular, much like the periphery of the meniscus in the knee [10]. The peripheral portions of the DP are thickened with longitudinally oriented collagen fibres, which travel between the radius and ulna in an axial orientation [10]. They can be identified sonographically. The arterial supply of the DP is provided mostly via dorsal and palmar branches of the anterior interosseous artery (AIOA), a branch of the common interosseous artery, which itself is a branch of the ulnar artery [14]. The AIOA may be seen sonographically leading to the DP; however, normal vessels within the DP itself are difficult to discriminate with colour or power Doppler. Increased vascularity within the peripheral portions of the TFCC may be appreciated sonographically when inflamed. The central (deeper) portion of the DP is relatively less vascular and thin, which predisposes it to perforation with age‐related degeneration [5, 10]. Degenerative changes in the DP develop as early as the fourth decade and progress with advancing age due to loss of normal collagen structure and increased water content [13].

Sonographically, the DP can be identified separately from the adjacent proximally located ulnar articular cartilage and distally located body of the MH. Differences in echogenicity between the DP and MH can be identified with altered angles of insonation. The DP is best sonographically assessed via a long axis imaging using both dorsal and ulnar approaches. When imaging the DP from a dorsal approach, wrist flexion and radial deviation can maximise sonographic visualisation at the radial sigmoid and ulnar foveal attachments, allowing more of the proximal lamina to be appreciated. Steep angulation of the transducer can allow the DP, as it courses along the curvature of the ulnar fovea, to be assessed.

When using an ulnar approach, the ECU is used as an acoustic window; this window is best to assess the ulnar styloid attachment of the distal lamina of the DP and differentiate it from the distally located MH. Ultrasound imaging is limited in demonstrating the central DP, particularly in the setting of positive ulnar variance where load through the centre of the DP to the ulnar head is greatly increased [14, 16]. Hence, ultrasound imaging cannot confidently and reliably exclude tears of the central DP and MR imaging is required for confirmation or exclusion of suspected tears of this region [14]. MR imaging also can demonstrate any associated chondromalacia of the lunate, triquetrum and distal ulnar head that may occur [14].

### 4.2 Meniscus Homologue

**FIGURE 6:**
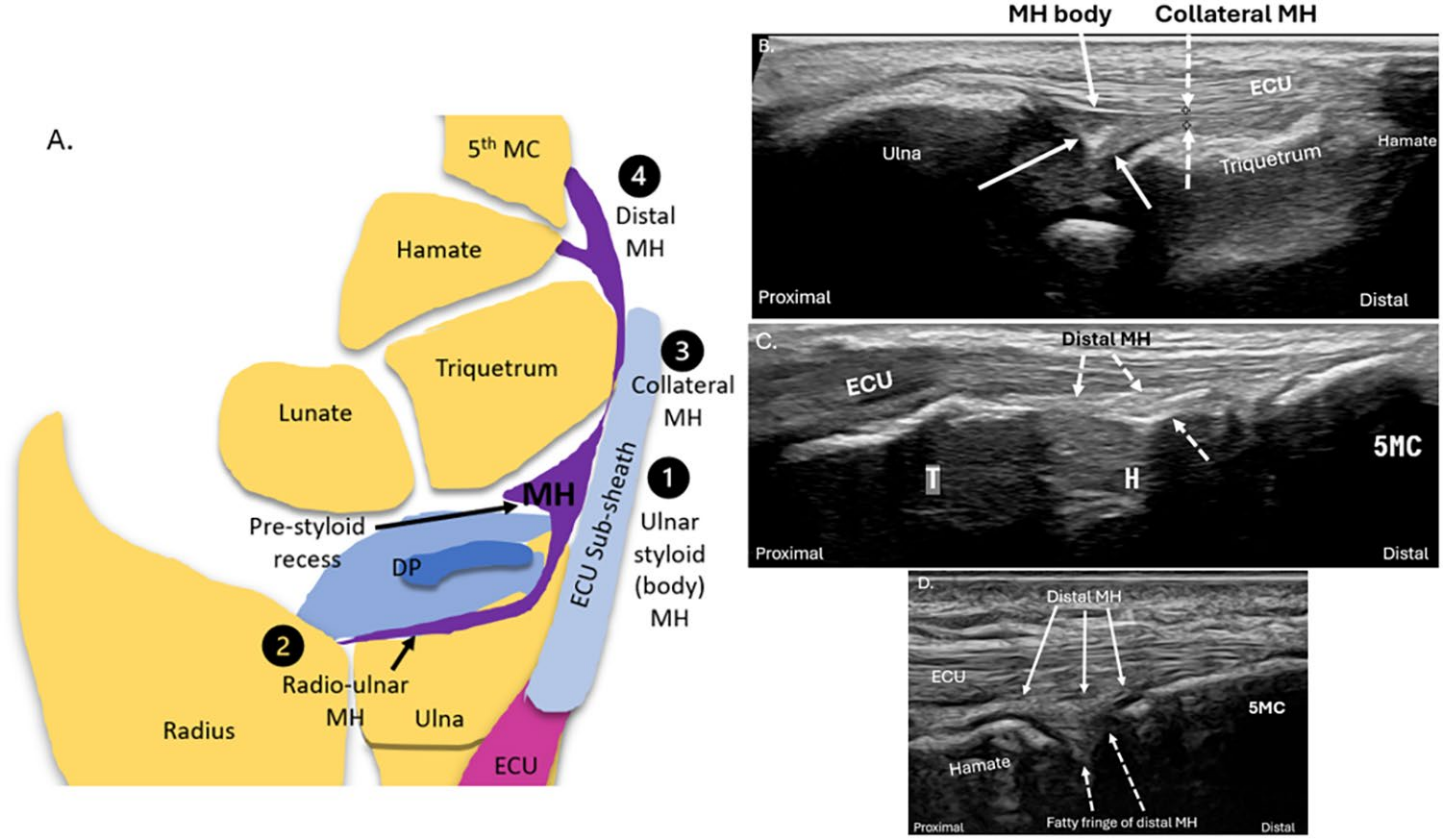
(A) Diagram of the meniscus homologue (MH) of the triangular fibrocartilage complex (TFCC) and the four parts of the MH which include the (1) ulnar styloid (body), (2) radio‐ulnar (dorsal), (3) collateral and (4) distal components. The pre‐styloid recess is a space where fluid can accumulate between the disc proper and the ulnar styloid (body) component of the MH. (B–D) Long axis sonographic images of the ulnar aspect of the MH. 5MC, 5th metacarpal; ECU, extensor carpi ulnaris; H, hamate; MC, metacarpal; T, triquetrum.

The meniscus homologue (MH), also termed the ulno‐meniscal homologue, is a complex sub‐structure of the TFCC located on the ulnar aspect of the TFCC which stabilises the distal ulno‐carpal space. It is commonly underappreciated; possibly due to it being inconsistently defined and described. The MH is divided into four parts: (1) the ulnar styloid (body), (2) radio‐ulnar (dorsal), (3) collateral and (4) distal components [5] (Figure 6).

The main component of the MH is formed by the ulnar styloid (body) component (1) which attaches to the ulnar styloid process [5]. Variations of the distal portion of the MH body attachment are described. An attachment to the proximal triquetrum is more common (90% population). In 10% of the population, the MH body can attach to the proximal triquetrum and extend to the proximal luno‐triquetral joint, piso‐triquetral joint or proximal lunate [17]. Other MH components are considered extensions of the MH body. The radio‐ulnar component (2) of the MH is the most proximal part; it blends with the DP and extends to the radius deep to the dorsal radioulnar ligament [17]. The collateral component (3) is thin and extends distal to the MH body, where it blends with the ECU sub‐sheath and ulnar collateral ligament; the three components cannot be distinguished on imaging at this point. The distal MH (4) extends to the ulnar aspect of the hamate and base of the 5th metacarpal [2, 17]. The distal component has fatty triangular deeper fringe elements which extend between the triquetrum and hamate and between the hamate and base of 5th metacarpal [13].

The MH is composed primarily of synovial tissue, which is well vascularised and innervated, differing from the fibrocartilaginous composition of the DP [5, 18]. This allows the MH to elongate, fold and stretch with radial deviation of the hand and supports the wrist during hyperflexion, along with the ECU sub‐sheath [5]. Due to its radio‐ulnar extension, the MH also acts as a shock absorber of the DRUJ [5]. The MH is inconsistently demonstrated on imaging and sonographically, the appearance of the MH is highly dependent on the wrist positioning during imaging [19]. Hence, tears of the deep portions of the MH may not be excluded sonographically [10].

As the MH is an ulnar‐sided structure, imaging in long axis from a dorso‐ulnar approach can be used to sonographically demonstrate the MH. When uninjured, the MH should be seen to slide dynamically with ultrasound imaging with radial and ulnar deviation. MH tears, when present, can occur to any of the four components of the MH and although they can occur in isolation, they more commonly occur concurrently with tears of other TFCC components [5]. Detachment of the MH entirely from the triquetrum can result in the Nishikawa‐tilt lesion [20].

#### 4.2.1 Ulnar Collateral Ligament

The ulnar collateral ligament (UCL) is a thin fibrous ligament that lies immediately superficial to the MH and deep to the ECU sub‐sheath; however, it is controversial as to whether the UCL is present or not [10]. It is not truly distinguishable from the MH or the ECU sub‐sheath on sonographic imaging. It is often described as the ‘ulnar capsule’ [10, 21]. The UCL is variably described as following the path of the MH, extending distally from the ulnar styloid process to the triquetrum, hamate and base of 5th metacarpal.

### 4.3 Pre‐Styloid Recess

Between the DP and the MH, is a triangular‐shaped but narrow synovial space referred to as the pre‐styloid recess, in which fluid can accumulate with varying wrist positions [2]. The function of the pre‐styloid recess is not well understood. Gas bubbles can be demonstrated moving within this space in asymptomatic wrists with dynamic sonographic imaging using radial deviation. The presence of fluid in this region and whether this finding indicates pathology requires further research (Video 2).

**VIDEO 2:** Demonstration of gas bubbles moving in pre‐styloid recess on long axis sonographic dynamic imaging. Video content can be viewed at https://onlinelibrary.wiley.com/doi/10.1002/ajum.70012

### 4.4 Extensor Carpi Ulnaris Sub‐Sheath

**FIGURE 7:**
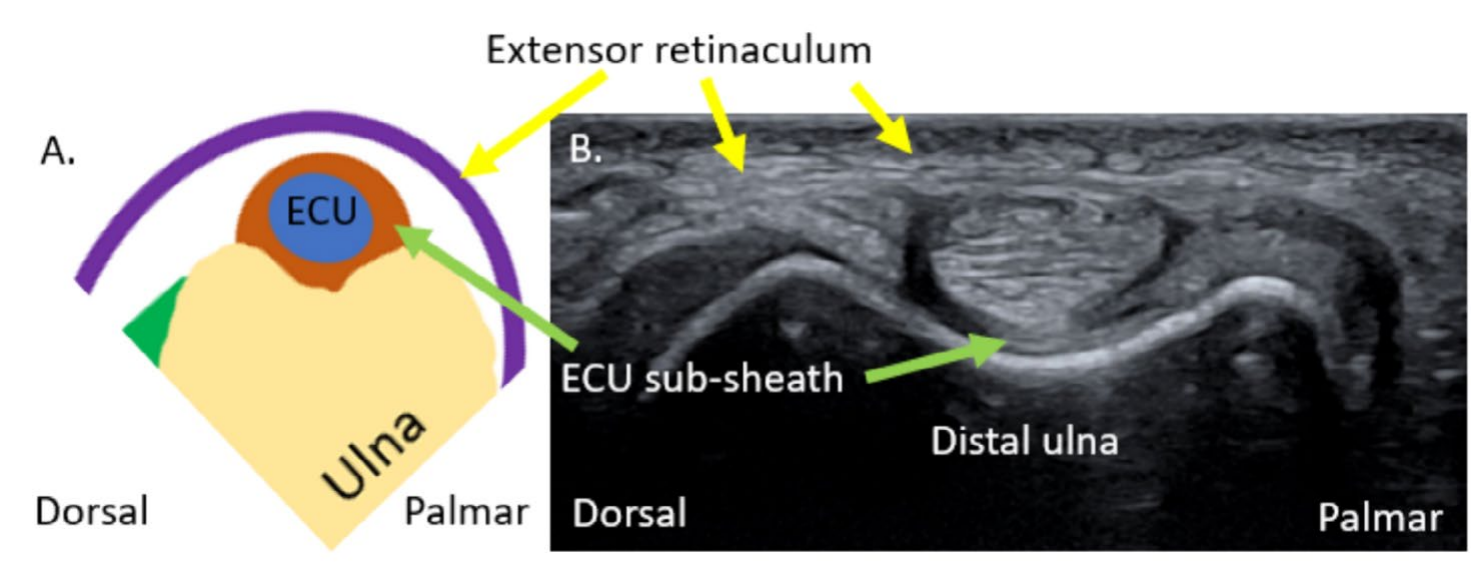
Extensor carpi ulnaris (ECU) sub‐sheath at level of distal ulna. (A) Diagram demonstrating the extensor retinaculum, superficial to the ECU tendon and sub‐sheath. (B) The ECU sub‐sheath surrounds the ECU tendon and adheres the ECU tendon to the ulna within the ulnar groove.

The ECU is a fusiform muscle in the posterior aspect of the forearm that spans from two proximal heads arising from the lateral epicondyle and middle third of the posterior ulna [10, 22]. The ECU functions to extend, adduct and ulnar deviate the hand. The ECU distal myotendinous junction is located at the level of the distal ulna. In the wrist, the ECU tendon courses over the dorso‐medial distal ulnar epiphysis, ulnar styloid, ulno‐carpal space, dorsal triquetrum and hamate, to insert onto the dorsal aspect of the 5th metacarpal base [5]. The ECU tendon has a sub‐sheath, a unique anatomical characteristic, which is an important stabiliser of the ulnar side of the TFCC [10]. There is a lack of consensus regarding the extent of the ECU sub‐sheath. It is described to form the ulnar (medial) border of the TFCC and blends with the DDRUL and is sometimes collectively considered the ‘functional ulnar collateral ligament’ of the wrist by providing minor stability to the DRUJ [5]. The ECU sub‐sheath can be described variably at different levels: (1) at the distal ulna, (2) superficial to the ulno‐carpal space, (3) overlying the triquetrum, hamate and base 5th metacarpal. The most consistent area it can be identified sonographically is at the level of the distal ulna where it appears as an echogenic band surrounding the ECU tendon. Further research is required to define it sonographically at more distal levels (Figure 7).

At the level of the distal ulna, the ECU tendon is enclosed by the ECU sub‐sheath. The ECU tendon and sub‐sheath are collectively held within the ulnar groove during rotation and flexion and extension of the wrist by the extensor retinaculum of the 6th dorsal wrist compartment [22]. At this level, the ECU sub‐sheath is anchored to the distal ulna, while the extensor retinaculum forms the roof of the osseous fibrous tunnel in which it sits, but the retinaculum does not have direct attachments to the ulna and prevents bowstringing of the ECU tendon during muscle contractions [5].

Distal to the ulna, and superficial to the MH overlying, the ECU sub‐sheath is not well defined anatomically, and there is a lack of consensus to its structure. At the level of the ulno‐carpal space, it is variably described as being formed by the merger of the deep aspect of the ECU tendon sheath, the ulnar collateral ligament (UCL) and the MH [5]. The ECU sub‐sheath changes in position between supination and pronation, and when the wrist is in a neutral position; it travels dorsally and medially to the ulnar styloid process, very close to and often indistinguishable from the ulnar collateral ligament [10]. Distally, at the level of the triquetrum, hamate and base of the 5th metacarpal, the ECU sub‐sheath becomes thinner.

### 4.5 Palmar Ulno‐Carpal Ligaments: ULL and UTL

**FIGURE 8:**
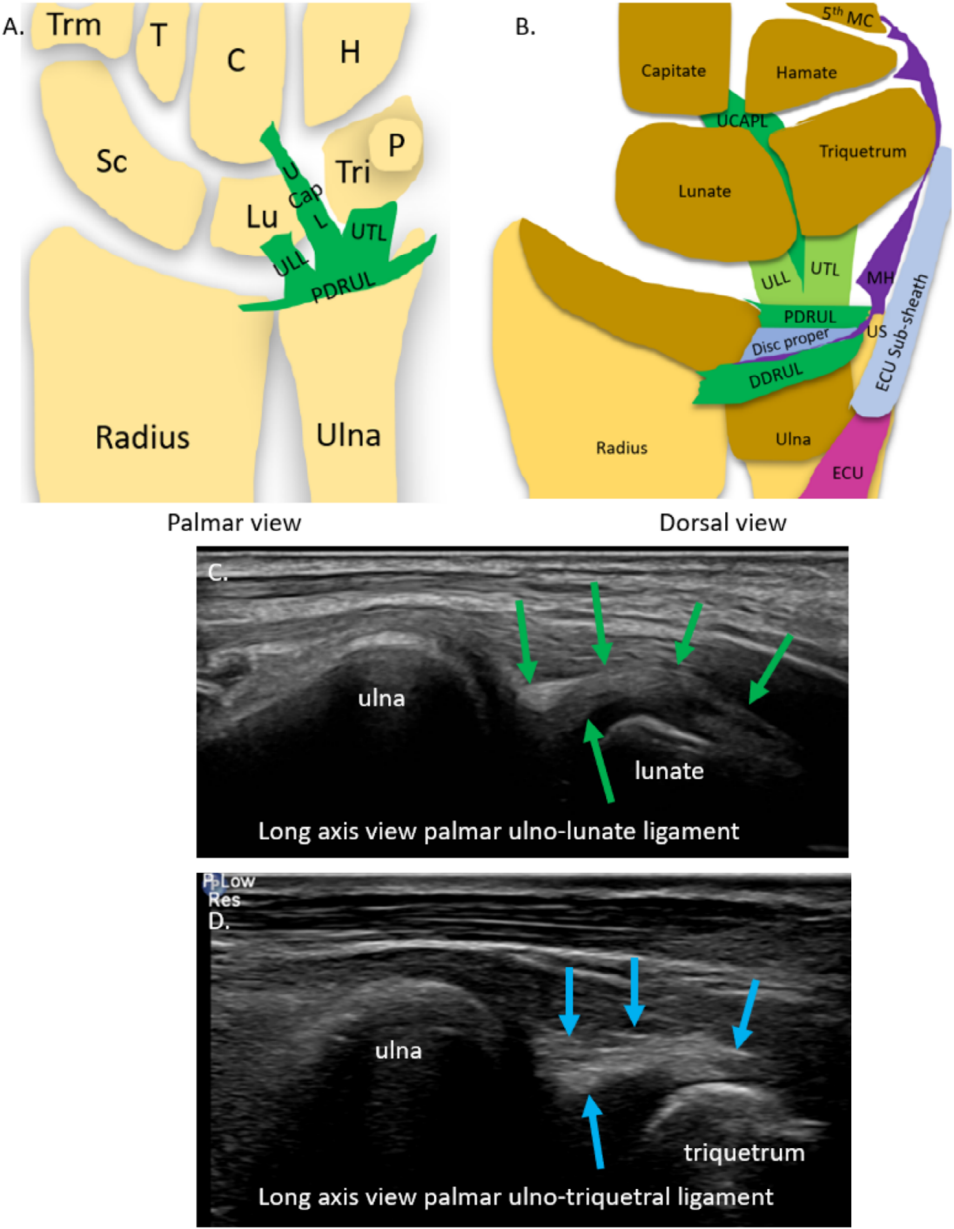
Palmar (A) and dorsal (B) view diagrams of the palmar ulno‐carpal ligaments which include the ulno‐lunate ligament (ULL), ulno‐capitate ligament (U Cap L) and the ulno‐triquetral ligament (UTL). These ligaments attach proximally to the palmar distal radio‐ulnar ligament (PDRUL) and fan out distally. (C, D) Associated sonographic long axis views demonstrating the palmar ulno‐lunate ligament (C) and palmar ulno‐triquetral ligament (D). C, capitate; DDRUL, dorsal distal radio‐ulnar ligament; ECU, extensor carpi ulnaris; H, hamate; Lu, lunate; MC, metacarpal; MH, meniscus homologue; P, pisiform; Sc, scaphoid; T, trapezoid; Tri, triquetrum; Trm, trapezium; US, ulnar styloid process.

The palmar aspect of the TFCC is composed of palmar deep extrinsic wrist ligaments called ulno‐carpal ligaments, which consist primarily of the palmar ulno‐lunate ligament (ULL) and the palmar ulno‐triquetral ligament (UTL). The palmar‐sided ULL and UTL provide additional support and reinforcement of the TFCC and contribute to DRUJ and ulnocarpal stability [5]. A further palmar ulno‐capitate ligament, which sits between the ULL and UTL, has been variably reported to constitute the TFCC; however, there is no consensus regarding its contribution to the TFCC and the stability it provides to the TFCC and DRUJ [5, 10]. Collectively these ligaments can be referred to as the ulno‐carpal ligamentous complex (UCLC) [10] (Figure 8).

The UCLC merges with the PDRUL. The ULL, UTL and ulno‐capitate ligaments arise from anchor points from the PDRUL at the fovea, base of the ulnar styloid and the DP and fan out distally to insert respectively onto the palmar cortex of the lunate, triquetrum and capitate [5, 10]. Additionally, the ULT has some fibres which attach to the palmar aspect of the MH. Sonographically, these ligaments are often confluent and indistinguishable from each other [10]. They can be demonstrated in their long axis by placing the transducer over the palmar ulnar aspect of the wrist between the distal ulna and triquetrum, lunate and capitate bones, which are used as sonographic landmarks to identify the ligaments deep to overlying flexor tendons. These ligaments can be torn during a TFCC injury and partial or complete tears of these ligaments can be demonstrated sonographically [23]. Partial tears will be identified by ligamentous thickening, hypoechogenicity and decreased echogenicity. The dorsal aspect of the ulno‐lunate and ulno‐triquetral joints are not outlined by specific ligaments as in the palmar wrist, and hence this is just referred to as the ‘dorsal capsule’.

### 4.6 Distal Radio‐Ulnar Ligaments: DDRUL and PDRUL

**FIGURE 9:**
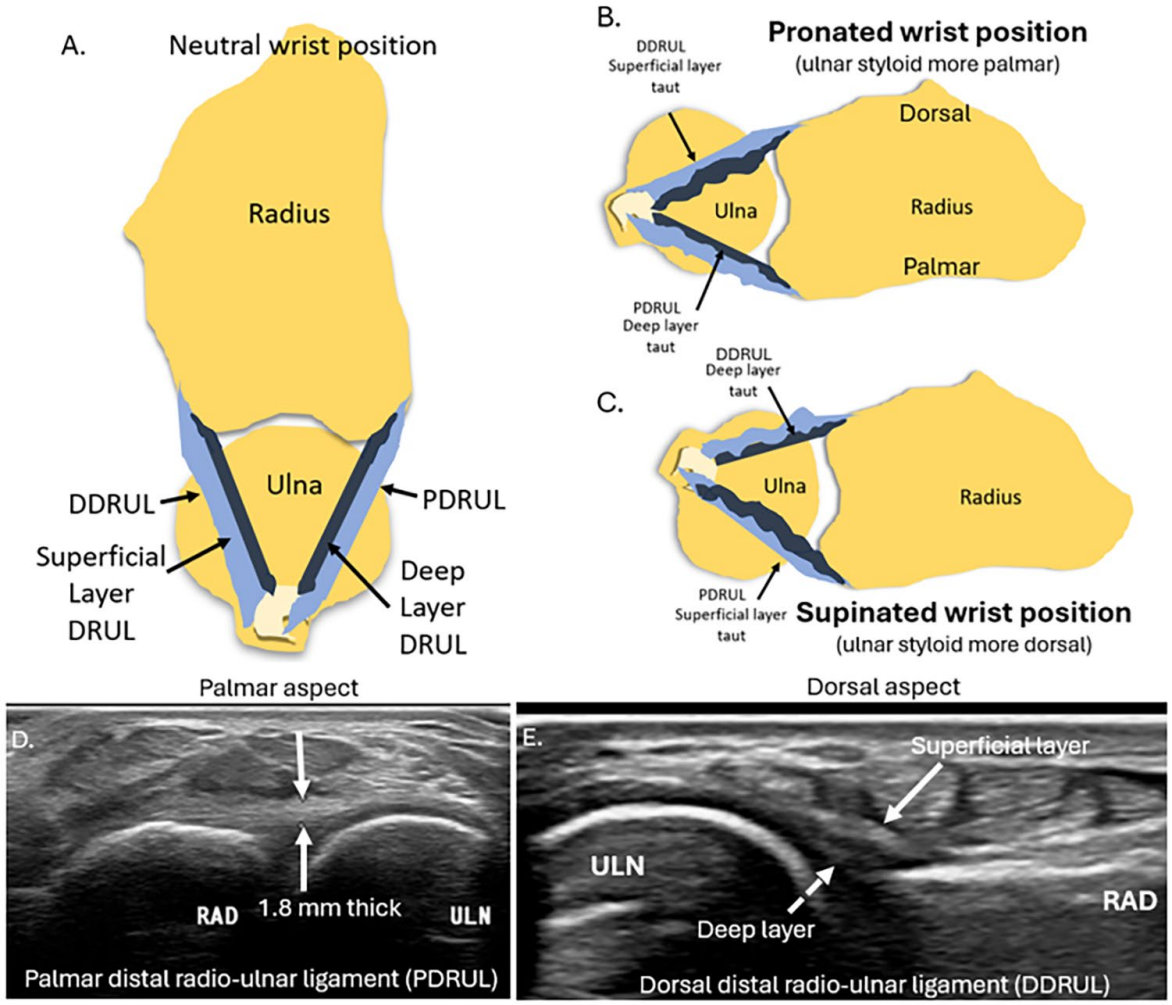
The dorsal and palmar distal radio‐ulnar ligaments become taut in different hand and wrist positions. (A) Hand and wrist neutral position. (B) Hand and wrist pronated. (C) Hand and wrist supinated. (D) Short axis sonographic view of the distal radio‐ulnar joint demonstrating the long axis of the palmar distal radio‐ulnar ligament (PDRUL) outlined by white arrows. (E) Long axis view of the dorsal distal radio‐ulnar ligament (DDRUL). The yellow arrow is pointing to the superficial layer, the green arrow is pointing to the deep layer of the DDRUL. RAD, radius; ULN, ulna; US, ulnar styloid process.

The distal radio‐ulnar ligaments consist of both the dorsal distal radio‐ulnar ligament (DDRUL) and the palmar distal radio‐ulnar ligament (PDRUL) [5]. They extend from the ulnar styloid and fovea to the dorsal and palmar rims of the sigmoid notch of the radius [24]. During pronation and supination, the radius rotates around the stationary ulna, and the DDRUL and PDRUL support the DRUJ and prevent subluxation of the ulnar head. The distal radio‐ulnar ligaments (DRULs) are composed of superficial and deep layers [5]. The superficial fibres insert at the ulnar styloid and the deep fibres insert into the ulnar fovea, providing a four‐armed arrangement at the ulnar aspect [5, 24]. The deep layers enclose the DP and blend with its periphery contributing to its increased thickness at the periphery [10, 24]. When the hand and wrist are pronated, the superficial DDRUL and deep PDRUL are taut, and on supination, the deep DDRUL and superficial PDRUL are taut [5] (Figure 9).

Sonographically, the DRULs are best imaged in the long axis, from a palmar and dorsal approach; the transducer is oriented perpendicular to the long axis of the distal radius and ulna [25]. The DRULs appear as fibrillar bands, and dynamic imaging with pronation and supination of the wrist can assist in better defining and assessing the ulnar attachments [25].

## 5 Comparing Ultrasound and Magnetic Resonance Imaging of the Triangular Fibrocartilage Complex

The TFCC is a complex structure, and tears to any of its individual components are a common source of ulnar‐sided wrist pain and can be demonstrated on both ultrasound and MR imaging [15, 19]. They can both play complementary roles in the evaluation of different causes of ulnar‐sided wrist pain and instability [15, 26]. Ultrasound imaging allows real‐time dynamic imaging, demonstrates the peripheral components of the TFCC well and can be used to guide the delivery of injectates for pain relief [25, 26]. MR arthrography demonstrates improved sensitivity and specificity in detecting and localising TFCC injuries, particularly when injuries involve the deep or central portion of the TFCC [26].

## 6 Conclusion

Ulnar sided wrist pain can be difficult to diagnose and treat. High resolution ultrasound can be used to image the ulnar side of the wrist, including the TFCC and DRUJ. An appreciation of the anatomical intertwining of structures forming the TFCC and the DRUJ, their normal sonographic appearances and the technique to image the ulnar wrist can enhance the quality of sonographic imaging. The limitations of ultrasound imaging in demonstrating the central portions of the TFCC must also be appreciated.

## Author Contributions

The authorship listing conforms with the journal's authorship policy and all authors are in agreement with the content of the submitted manuscript.

## Ethics Statement

Human Research ethics committee approval and clearance was not required for the completion of this review paper.

## Conflicts of Interest

The authors declare no conflicts of interest.
